# Common haematological malignancies in Northeastern Nigeria: a multi-centre study of their pattern, distribution and treatment challenges

**DOI:** 10.3389/fonc.2025.1404686

**Published:** 2025-03-31

**Authors:** Rufai Abdu Dachi, Falmata Grema Mustapha, Jesini James, Kasim Muhammad Pindiga, Uchenna Simon Ezenkwa, Maimuna Orahachi Yusuf, Saleh Yuguda, Dauda Eneyamire Suleiman

**Affiliations:** ^1^ Department of Haematology and Blood Transfusion, Abubakar Tafawa Balewa University, Bauchi, Bauchi, Nigeria; ^2^ Department of Haematology and Blood Transfusion, Modibbo Adama University Teaching Hospital, Yola, Adamawa, Nigeria; ^3^ Department of Haematology and Blood Transfusion, Federal Teaching Hospital, Gombe, Gombe, Nigeria; ^4^ Department of Pathology, Federal University of Health Sciences, Azare, Bauchi, Nigeria; ^5^ Department of Paediatrics, Federal University of Health, Azare, Bauchi, Nigeria; ^6^ Department of Haematology and Blood Transfusion, Gombe State University, Gombe, Nigeria; ^7^ Department of Histopathology, College of Medical Sciences, Abubakar Tafawa Balewa University, Bauchi, Bauchi, Nigeria

**Keywords:** haematological malignancies, patterns, distribution, challenges, Northeastern Nigeria

## Abstract

**Background:**

Haematological malignancies (HMs) are primary cancers of the blood and blood-forming organs. They are heterogeneous and of diverse clinical features, treatment protocols and prognoses. They constitute a significant source of cancer-related morbidity and mortality. Northeastern Nigeria, being the region in the country with the worst literacy rate and poverty indices, is also battling with the burden of these diseases with the limited health facilities to adequately diagnose and treat these ailments. There is paucity of a comprehensive data on HMs in the region, so this study aims to report on the multi-centre burden of the common HMs in the region and to discuss their patterns of distribution and management challenges.

**Materials and methods:**

This was a 5-year retrospective study where records of cases of HMs diagnosed in the four health facilities [viz., Abubakar Tafawa Balewa University Teaching Hospital (ATBUTH), Bauchi; Federal Teaching Hospital (FTH), Gombe; Modibbo Adama University Teaching Hospital (MAUTH), Yola; and Federal Medical Centre (FMC), Azare] were collected from 1 January 2018 to 31 December 2022. Data on the age, gender, diagnosis and subtypes of some malignancies were also obtained and analysed using the SPSS Version 23.0 statistical software.

**Results:**

A total of 493 cases of HMs, which constituted 8.2% of all cancers, were diagnosed during the period under review. Paediatric HMs constituted 42.0% (207/493) of the HMs. Non-Hodgkin lymphoma (NHL) constituted the majority of the HMs at 115/490 (23.5%), while multiple myeloma (MM) was the least at 38/493 (7.7%). An average cost of 5,000 to 10,000 United States dollars is required to manage an HM in Nigeria. Late presentation of patients, non-availability and inadequate number of personnel, inability to pay for investigations and/or treatments due to financial constraints, and limited facilities for tests such as flow cytometry, immunohistochemistry, cytogenetic and molecular genetic analyses were the challenges identified in the institutions in Northeastern Nigeria that manage cancer patients.

**Conclusion:**

Haematological malignancies are common in our environment, and there are limited facilities and expertise to accurately diagnose and treat them in the region and Nigeria in general.

## Introduction

Haematological malignancies (HMs) are primary clonal disorders of the blood and blood-forming organs that are characterized by abnormal proliferation and accumulation of malignant cells in various body tissues and organs (1). They are a group of heterogeneous and diverse diseases composed of infiltrates of mature or immature haemopoietic cells at various stages of differentiation, along the lymphoid and myeloid lineages. Significant variations exist in their incidences, biological behaviours, clinical presentations, treatment options and prognosis (2). The clinical presentations of HMs vary between diseases, and these include features of bone marrow failure (such as anaemia, neutropaenia and thrombocytopenia) and organ infiltrations, presenting with organomegaly, i.e., lymphadenopathy, splenomegaly and hepatomegaly. They can sometimes infiltrate other organs like the central nervous system, testes and eyes, presenting with features related to these organs (3). These diseases include leukaemias [which can be acute myeloid leukaemia (AML), acute lymphoblastic leukaemia (ALL), chronic myeloid leukaemia (CML) or chronic lymphocytic leukaemia (CLL)], lymphomas (Hodgkin and non-Hodgkin types), multiple myeloma (MM), myelodysplastic syndrome (MDS) and myeloproliferative neoplasms (which include polycythaemia rubra vera, essential thrombocythaemia and myelofibrosis) (3).

Haematological malignancies, like most other cancers, largely have unknown aetiologies. However, strong relationships have been established between the role of genetic defects such as cytogenetic abnormalities, gene mutations and abnormal gene expression as well as environmental exposure to some carcinogens (chemicals such as petro- and agro-chemicals and some radiological substances) in the pathogenesis of these malignancies (4). HMs constitute serious public health challenges in both developed and developing countries due to their associations with increased morbidity and mortality, with the burden being more pronounced in low- and middle-income countries (LMICs) like Nigeria owing to the high frequency of late presentation of cases as well as the inadequacy of diagnostic and therapeutic facilities in addition to a serious financial burden where patients pay out of pocket for all health-related needs (5).

Population-based incidence and survival outcomes of HMs in many sub-Saharan African countries have been difficult to estimate for many years. Lack of functional cancer registries and/or rudimentary or non-existent medical records are partly responsible for the partial information about cancer incidence, treatment and follow-up in Africa (6). However, the global picture reveals that HMs represent approximately 6.5% of all cancers worldwide, and they are the fourth most frequently diagnosed cancers in both men and women in developed countries of the world (7). The breakdown of this figure shows that NHL accounts for 2.7%, while leukaemias, multiple myeloma and HL represent 2.5%, 1.0% and 0.8%, respectively (8). It is of note that the incidences are on the rise globally, owing to improved understanding of the biology of the tumours and improved diagnostic facilities where hitherto inaccessible diagnostic modalities such as cytogenetic analyses and molecular diagnostics are now conducted frequently in many parts of the world, including in developing countries. According to the World Health Organization (WHO) 5th Edition of Classification of Haematolymphoid Tumours, they are classified broadly into myeloid, lymphoid, histiocytic/dendritic cell and stroma-derived neoplasms of lymphoid tissues.

A systematic review by Ugwu et al. revealed that challenges in diagnosing and managing haematological malignancies encompass late patient presentation at advanced disease stages, diagnostic difficulties or misdiagnosis stemming from reliance solely on morphology for histological diagnosis without ancillary immunohistochemistry, financial barriers preventing access to investigations and treatment due to the high cost of cytotoxic drugs and poverty-related constraints, and the absence of adequately equipped facilities for the management of HMs (7).

As a consequence of the challenges highlighted in the study above, the health outcomes of the management of HMs in Nigeria were observed generally to be poor and characterized by needless mortality, incomplete treatment and follow-up, and discharges against medical advice largely due to socio-economic challenges (7).

There is paucity of a comprehensive data and information about patterns of haematological malignancies in Northeastern Nigeria. However, there are few centre-based reports from Yola, Bauchi and Maiduguri, where varying proportions of HMs were reported (8–10). This study aims to report the pattern and distribution of the common haematological malignancies in multiple centres from Northeastern Nigeria where specialist haematological and histopathological services exist with expertise to diagnose haematological malignancies via bone marrow aspiration cytology, trephine biopsies and histopathological examination of solid haematological tumours such as lymphomas.

## Materials and methods

This was a 5-year retrospective study where records of cases of haematological malignancies diagnosed in the Haematology and Histopathology Departments of Abubakar Tafawa Balewa University Teaching Hospital (ATBUTH), Bauchi; Federal Teaching Hospital (FTH), Gombe; Modibbo Adama University Teaching Hospital (MAUTH), Yola; and Federal Medical Centre (FMC), Azare, were obtained from the Bone Marrow Aspiration cytology register as well as the cancer registers of the various pathology departments from 1 January 2018 to 31 December 2022. Data on the age, gender, diagnosis and subtypes of some malignancies diagnosed were also collected. The collated data were analysed using SPSS Version 23.0 (IBM SPSS Statistics). Continuous variables with z-scores within ±2.58 for skewness and kurtosis were considered to be normally distributed. Normally distributed data were reported as means ± standard deviations (SDs), while non-normally distributed data were reported as median (interquartile ranges). Categorical data were reported as percentages. A p-value of ≤0.05 was considered significant.

## Results

**Figure 1:**
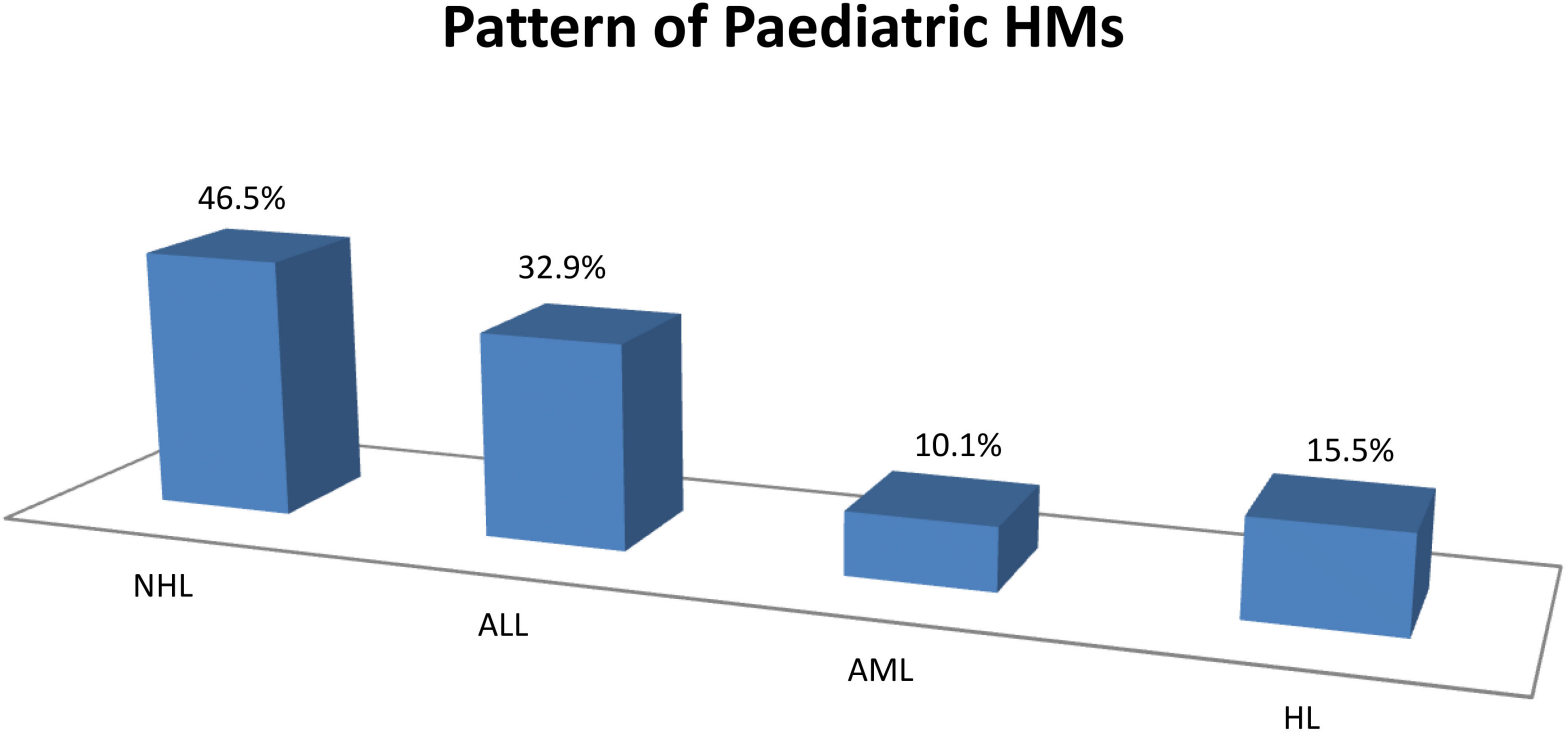
Distribution of the paediatric haematological malignancies.

**Figure 2:**
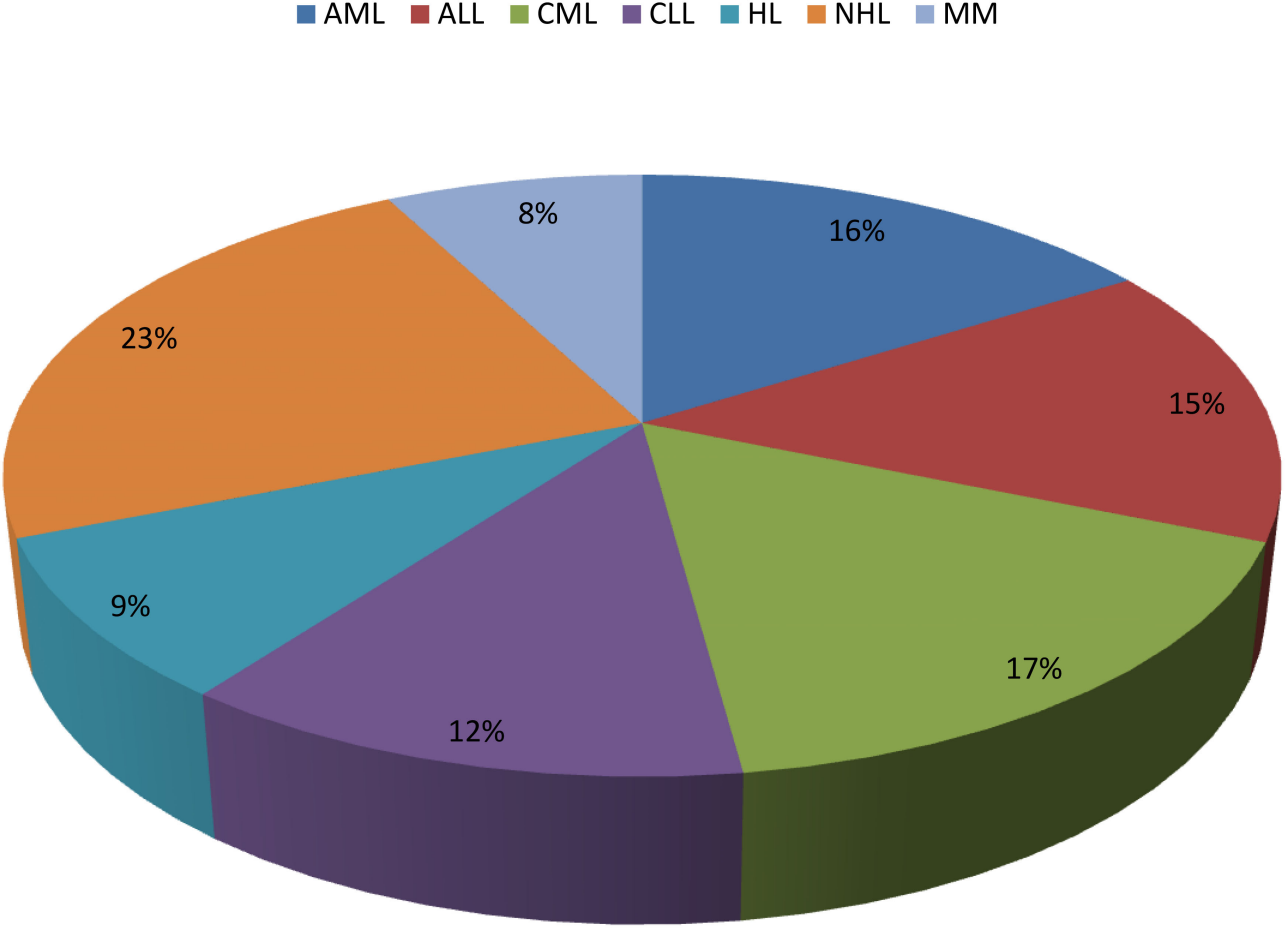
Distribution of haematological malignancies in Northeastern Nigeria.

A total of 493 cases of HMs were diagnosed during the period under review. They constitute 8.2% of all cancers diagnosed during the period. The ages of the participants range from 6 months to 80 years. A total of 207 paediatric haematological malignancies occurring in patients ≤15 years were recorded, representing 42.0% of the HMs observed during the period under review. The mean ages ± SD and gender distributions of the participants are shown in **Table 1**. For the paediatric haematological malignancies, non-Hodgkin lymphoma constituted the majority at 86/207 (41.5%), while AML was the least in prevalence, accounting for 21/207 (10.1%) as shown in **Figure 1**. For the generality of the HMs comprising both paediatrics and adult groups, non-Hodgkin lymphoma (NHL) constituted the majority of the HMs at 115/490 (23.5%), while MM was the least at 38/493 (7.7%) as shown in **Figure 2**. The distribution of the various HMs per health facility is presented in **Table 2**. An average cost of 5,000 to 10,000 United States dollars is required to manage an HM in Nigeria. Late presentation of patients to the health facilities to access care, non-availability and inadequate number of personnel to appropriately manage HMs, inability to pay for investigations and/or treatments due to financial constraints, as payment is mostly out of pocket and only a few of them have insurance coverage, and delay in arriving at a diagnosis due to limited facilities for further tests (such as flow cytometry, immunohistochemistry, cytogenetic and molecular genetic analysis) as well as poorly equipped health facilities to appropriately manage the conditions are the challenges identified across health institutions in Northeastern Nigeria.

**Table 1:** Distribution of participants’ age, gender and male–female ratio.

Haematological malignancy	Mean age ± SD (years)	Age range	Male–female ratio
AML	27.1 ± 9.2	12–58	2:1
ALL	16.8 ± 10.4	0.5–53	2.5:1
CML	55.0 ± 11.2	37–70	1:1
CLL	57.6 ± 18.2	50–80	1.5:1
MM	48.4 ± 9.5	45–60	1:1
NHL	22.8 ± 7.5	5–60	1.8:1
HL	21.0 ± 4.8	10–60	1.7:1

Key: AML, acute myeloid leukaemia; ALL, acute lymphoblastic leukaemia; CML, chronic myeloid leukaemia; CLL, chronic lymphocytic leukaemia; NHL, non-Hodgkin lymphoma; HL: Hodgkin lymphoma.

**Table 2:** Distribution of the various haematological malignancies per health facility.

Health facility	AML	ALL	CML	CLL	HL	NHL	MM
ATBUTH	22	27	23	29	13	36	11
FTHG	31	22	24	14	18	38	14
MAUTH	23	18	29	16	8	34	13
FMCA	3	8	6	2	4	7	0
Total	79	75	82	61	43	115	38

Key: AML, acute myeloid leukaemia; ALL, acute lymphoblastic leukaemia; CML, chronic myeloid leukaemia; CLL, chronic lymphocytic leukaemia; NHL, non-Hodgkin lymphoma; HL, Hodgkin lymphoma; ATBUTH, Abubakar Tafawa Balewa University Teaching Hospital; FTHG, Federal Teaching Hospital, Gombe; MAUTH, Modibbo Adama University Teaching Hospital; FMCA, Federal Medical Centre, Azare.

## Discussion

Northeastern Nigeria comprises six states, viz., Adamawa, Bauchi, Borno, Gombe, Taraba and Yobe states, and is home to approximately 26 million people, representing 12% of Nigeria’s population (11). The zone has nine tertiary health facilities, but only five have functional specialist haematological services that have the capacity to diagnose and treat HMs. These include ATBUTH, Bauchi; FMC, Azare; MAUTH, Yola; FTH, Gombe; and University of Maiduguri Teaching Hospital (UMTH) Maiduguri, so the data here were from these centres as presented in the Results section. This study recorded 493 cases of haematological malignancies during the period of study, which represent 8.2% of all cancers diagnosed. This is similar to what was reported by Babatunde et al. and Errahhali et al., who respectively reported 8.1% and 9.3% cases of HMs in Ilorin, North Central Nigeria and Eastern Morocco (12, 13).

The age range of the study participants was between 6 months and 80 years. This indicates that HMs just like other malignancies can be found among all age groups. The result is similar to what was reported by Babatunde et al., who reported an age range of 7 months to 80 years (12). A contrasting finding was reported by Akaba et al. in Calabar, south–south Nigeria, where an age range of 20–89 years was observed (14).

This study showed that HMs occurred more frequently in male than female individuals in this region. This is similar to reports of HMs by Babatunde et al., Errahhali et al. and Akaba et al. in Ilorin Nigeria, Eastern Morocco and Calabar, south–south Nigeria, respectively (12–14). A contrasting result of female preponderance was reported by Perez et al. in Chile (15). The high male preponderance in our study is similar to most publications on HMs both nationally and internationally. This could be attributed to increased exposure to potentially carcinogenic occupational and environmental agents (14). There are different types of HM, and this study showed that the majority of the patients had NHL. This is similar to reports by Akaba et al., Perez et al. and Hungria et al. in Nigeria, Chile and Latin America (14–16). Multiple myeloma was reported to be the least of all the HMs in this study, and it is similar to what was reported by Perez et al.; however, Hungria et al. reported contrasting results, where they found CLL as the least of the HMs (15, 16).

The challenges of diagnosis and management of HMs are common to almost all the centres in Nigeria, as previously reported by Dachi et al. and Ugwu et al., where issues such as late presentation of the patients in the advanced stage of the disease, inability to pay for investigations and/or treatment due to financial constraint, and wrong or delayed diagnosis due to inadequate diagnostic facilities, as most centres mainly rely only on morphologic appearance for histological diagnosis without immunohistochemistry. Poorly equipped health facilities for the management of haematological malignancies equally contribute to poor outcomes in these patients (4, 7). Delayed presentation, which can be due to ignorance about the disease among patients due to low literacy rates, has also been a source of worry. Financial constraints in settling the bills of investigations and treatment of cancers, in general, and haematological malignancies, in particular, is also a big challenge in many African countries (17–19).

The challenges can be tackled by awareness creation and education on the importance of early presentation to health facilities; health systems should be strengthened by including HMs in the coverage of the National Health Insurance and adequately equipping health facilities for proper diagnosis and management of HMs.

There are unexplored areas in relation to HMs in Northeastern Nigeria that can be further explored for future research. These include the following:

- Are there differences in tumour biology and clinical differences between the different HMs in Northeastern Nigeria and other parts of the world?
- What are the barriers and facilitators to accessing standard-of-care treatments in patients with HMs in Northeastern Nigeria?
- What is the effect of HMs on the quality of life of patients in Northeastern Nigeria?

## Conclusion

Haematological malignancies are common in our environment, and there are limited facilities and expertise for the comprehensive management of these patients, not only in the northeast region but in Nigeria in general. There are also some research areas that need to be looked into to fully characterize and adequately manage HMs in the region. This can be conducted by building the capacity of staff and establishing robust centres that can provide holistic care to oncology patients.

### Recommendations

Collaborative work with experts in the management of haematological malignancies here in Nigeria and other parts will surely add value to both the diagnosis and treatments of these diseases and avert the observed challenges, so these are strongly recommended.

## Data Availability

The raw data supporting the conclusions of this article will be made available by the authors, without undue reservation.
